# Quantification of Nonproteolytic Clostridium botulinum Spore Loads in Food Materials

**DOI:** 10.1128/AEM.03630-15

**Published:** 2016-03-07

**Authors:** Gary C. Barker, Pradeep K. Malakar, June Plowman, Michael W. Peck

**Affiliations:** Institute of Food Research, Norwich Research Park, Colney, Norwich, United Kingdom; Rutgers, The State University of New Jersey

## Abstract

We have produced data and developed analysis to build representations for the concentration of spores of nonproteolytic Clostridium botulinum in materials that are used during the manufacture of minimally processed chilled foods in the United Kingdom. Food materials are categorized into homogenous groups which include meat, fish, shellfish, cereals, fresh plant material, dairy liquid, dairy nonliquid, mushroom and fungi, and dried herbs and spices. Models are constructed in a Bayesian framework and represent a combination of information from a literature survey of spore loads from positive-control experiments that establish a detection limit and from dedicated microbiological tests for real food materials. The detection of nonproteolytic C. botulinum employed an optimized protocol that combines selective enrichment culture with multiplex PCR, and the majority of tests on food materials were negative. Posterior beliefs about spore loads center on a concentration range of 1 to 10 spores kg^−1^. Posterior beliefs for larger spore loads were most significant for dried herbs and spices and were most sensitive to the detailed results from control experiments. Probability distributions for spore loads are represented in a convenient form that can be used for numerical analysis and risk assessments.

## INTRODUCTION

Minimally processed chilled foods, or partially processed chilled foods, are foods that have fresh-food-like characteristics and satisfy consumer demand for foods that are mildly heated, without added preservatives, higher in nutritional value, and easy to prepare (1–3). These foods form an increasing part of the diet in many parts of Europe, and in the United Kingdom, the chilled food sector employs a substantial fraction of the food manufacturing workforce. Estimates indicate that in the United Kingdom in 2014, the value of the market for chilled prepared food exceeded £12 billion (http://www.chilledfood.org/MEDIA).

Clostridium botulinum is a diverse species that comprises four distinct groups of bacteria, all of which form botulinum neurotoxin. There are seven types of botulinum neurotoxin (A to G), and it is the most potent toxin known, with as little as 30 to 100 ng of neurotoxin potentially being fatal (4–7). Proteolytic C. botulinum (group I) and nonproteolytic C. botulinum (group II) are associated with food-borne botulism. Strains of proteolytic C. botulinum form neurotoxin of types A, B, and/or F, form very heat-resistant spores, and have a minimum growth temperature of 10°C to 12°C, while strains of nonproteolytic C. botulinum form neurotoxin of type B, E, or F, form spores of moderate heat resistance, and multiply and form neurotoxin at 3.0°C to 3.3°C in 5 to 7 weeks but not at 2.5°C or below in 12 weeks (4–6, 8–10). Nonproteolytic C. botulinum has been identified as a hazard in minimally processed chilled foods. Mild thermal processing conditions cannot guarantee complete inactivation of spores, and typical refrigeration conditions may permit spore germination, population growth, and toxin production by nonproteolytic C. botulinum. The consumption of even small amounts of preformed botulinum neurotoxin in food can cause severe illness and potentially death. The safety of these foods relies on a combination of good-quality raw materials, mild heat treatment, manufacturing hygiene, chilled storage, and a restricted shelf life (1, 2). The safety record for mass-produced minimally processed chilled food is very strong, but there have been occasional incidents that have involved time and/or temperature abuse of the final product or home-prepared foods (1, 2, 8, 11, 12). These sporadic incidents drive exceptional vigilance.

A crucial piece of information in quantifying the risk from nonproteolytic C. botulinum concerns the initial spore loads in food materials, but detection of these spores is particularly challenging. Currently the optimized protocol developed by Peck et al. (13) is considered a very sensitive enumeration method for spores of nonproteolytic C. botulinum in food materials. With this method, ∼400 spores kg^−1^ were observed in scallops. However, loads typically are smaller, and ∼1 spore kg^−1^ could be considered a significant contamination level for food materials (14). As a contribution to risk assessment, an effective quantification of spore loads for nonproteolytic C. botulinum must embrace the large variations in concentration but importantly must also represent uncertainties, such as those associated with the experimental detection limit (15).

For nonproteolytic C. botulinum, the origins of spore contamination in food materials are unclear. Spores are ubiquitous in the environment, but the steps and processes leading to food contamination have not been identified explicitly. In addition, the actual loads in food materials generally are very small and very difficult to detect, so that observations failing to detect spores predominate. Together, these factors ensure that the statistical properties of nonproteolytic C. botulinum spore loads in food materials, variability and uncertainty, are not well established. In many respects a Bayesian approach, using gathered evidence to build on broad prior beliefs, is suited to address this problem. Crépet et al. (16) employed a Bayesian scheme to build a representation for the contamination of fresh vegetables with Listeria monocytogenes, based on distinct observations describing both concentration and prevalence. Gonzales-Barron et al. (17) used a Bayesian framework to model belief concerning the contamination of sausages by Salmonella enterica serovar Typhimurium, and Daelman et al. (18) report a Bayesian model for Bacillus cereus spore contamination of food materials that are used in the production of minimally processed chilled foods. Alternative but comparable approaches for estimating probability distributions that represent bacterial contamination in food, based on maximum likelihood, have been reported by Busschaert et al. (19) and by Pouillot et al. (20); both studies concentrate on Listeria contamination.

Here, we develop a quantitative representation of beliefs concerning the concentration of nonproteolytic C. botulinum spores in a range of food materials that are used for manufacturing minimally processed chilled foods in the United Kingdom. Initial steps correspond with a literature survey, and the formation of a knowledge base, of reported spore loads in foods. In turn, this information is aggregated in a probabilistic representation expressing the variability and uncertainty of spore loads in nine representative food categories. Finally, evidence from a large set of controlled laboratory observations of real food materials is used to update beliefs about spore concentrations using a Bayesian approach. The posterior distribution of spore loads represents quantitative beliefs and currently gives the best support for the assessment of risks.

## MATERIALS AND METHODS

### Literature survey for information concerning spore loads in food materials.

Information about C. botulinum spore loads in food materials was accessed from a variety of sources, including scientific journals, books, technical reports, and expert opinions. A search in scientific databases, such as the Thomson Reuters Web of Science and PubMed (based on key words), as well as contributions from experts with personal document collections and from the Institute of Food Research botulinum database, identified more than 700 appropriate references (we thank B. M. Lund and T. A. Roberts for allowing access to their collections of papers on C. botulinum). Foreign-language reports were included in the list when practical. The initial list was filtered, by a microbiologist, to recover 100 sources that could contribute primary evidence on spore concentration (i.e., not reviews or summarized data). These sources are included as a comma-separated variable file in the supplemental material.

Most experimental investigations included enrichment in microbiological media followed by the mouse test or enzyme-linked immunosorbent assay (ELISA) to detect botulinum neurotoxin or PCR to detect the neurotoxin gene. In many cases, the reported presence of type B or F toxin did not allow differentiation between proteolytic C. botulinum and nonproteolytic C. botulinum. For those records, either was assumed to be present.

Primary data on the origin of materials, the food type, the experimental methods, the microbiology (including type information), and the results were systematically extracted from these sources and entered into a database with approximately 30 fields. The selected sources contributed approximately 1,200 records to the database. Each record corresponds to a single test applied to a particular food material. Expert microbiologists scored each record, on a scale of 1 to 5, according to the quality of the microbiological method. The details of the scoring system are presented in Table 1.

**TABLE 1:** Scoring system for microbial methods used to prioritize records for the food materials C. botulinum database

Score	Description
1	Not adequately described
2	Can be clearly identified as unlikely to recover nonproteolytic C. botulinum spores, e.g., where the heat treatment included temperatures of ≥80°C; the use of such a heat treatment to inactivate competing vegetative bacteria is valuable for isolation of proteolytic C. botulinum spores but may inactivate nonproteolytic C. botulinum spores
3	Can recover most nonproteolytic C. botulinum spores but is suboptimal, e.g., incubation at 35–37°C, weak anaerobically, or trypsin was not used in the mouse test; optimum growth temp for nonproteolytic C. botulinum is 25–30°C, and that of proteolytic C. botulinum is 37°C; toxins of nonproteolytic C. botulinum may not exhibit maximum toxicity until they are activated by trypsin
4	Suitable for recovering nonproteolytic C. botulinum spores but is not fully quantified, e.g., the detection limit is not determined; if samples are positive, an MPN technique should be included to estimate the numbers present
5	Suitable for recovering and quantifying nonproteolytic C. botulinum spores and includes controls; to calculate the detection limit for each food material, parallel samples should be inoculated with spores from mixtures of nonproteolytic C. botulinum strains and subjected to the same procedure

For each data record that has a quality score above two, i.e., 1,089 records, an estimate of spore concentration and an associated standard error were constructed by using a most-probable-number (MPN) calculation (21) based on the reported number of tests, the number of positive tests, and the quantity of material tested (for records where all tests are negative, a single positive test result for the most sensitive test was added to obtain a conservative MPN estimate; e.g., see Carlin et al. [22]). Note that for food materials we use volume (*V*) and mass interchangeably. The database represents an extensive source of information on spore loads for nonproteolytic C. botulinum in food materials. The database description and the details of spore loads are included as comma-separated variable files in the supplemental material.

### Categorization of food materials.

Commercial minimally processed chilled foods are constructed from a wide variety of raw materials and ingredients, often from many different production systems and from many different locations, so that it is impractical to establish statistical beliefs concerning the nonproteolytic C. botulinum spore loads for each individual food product. However, reports of active surveillance and expressed opinions suggest that the aggregation of spore load information, using a relatively small number of food groups or categories, can successfully represent beliefs about the contamination levels in primary food materials.

An expert group consisting of microbiologists, modelers, and specialists involved in the manufacture of minimally processed chilled foods provided guidance on the organization of spore load information. The experts suggested nine distinct, aggregated groups of food materials to support a quantitative representation of nonproteolytic C. botulinum spore load information: meat (ME), fish (FI), shellfish (SH), cereals, including pasta and rice (CE), fresh plant material, including fresh herbs (PL), dairy liquid (DL), dairy nonliquid (DN), mushroom and fungi (MF), and dried herbs and spices (HS). The categorization matched the needs of the manufacturers of chilled foods (in the United Kingdom) and also took into account the distribution of available data across food categories. Thus, although spore loads are believed to be high in honey (14), manufacturers of chilled thermally processed foods rarely include honey as an ingredient, so it is excluded from the categorization scheme. Similarly, information concerning spore loads in oils and fats is unavailable, so these materials are excluded from the assessment. Two hundred seven records from the database do not fit into the categorization scheme that represents materials used in minimally processed chilled foods in the United Kingdom. Carlin et al. (22) used a similar aggregation of foods in their survey of C. botulinum in raw food materials used in minimally processed chilled foods in France, and Daelman et al. (18) reported the use of five food groups to represent B. cereus spore contamination in materials used for the production of minimally processed chilled foods in Belgium.

### Variability and uncertainty for C. botulinum spore loads in food materials.

A representation for C. botulinum spore contamination in food materials begins with the spore concentration, *s* (per kilogram). In foods, C. botulinum spore loads are very small (typically an *s* of ∼1 kg^−1^) so that concentration is well defined (i.e., it is a continuous variable) only in relatively large volumes (i.e., *V* ≫ 1 kg). In this case, a (batch) processing volume, typically 10^3^ kg, can be identified as the primary element for consideration. A batch is often identified uniquely for product tracing purposes. In this representation, each batch is considered homogenous so that the contamination of smaller volumes can be considered in terms of independent random samples, but the contamination of batches from a single food category is variable. This contamination structure, expressing within-batch variability as small compared to between-batch variability, is consistent with the aggregation property of the food material categorization scheme and with the stochastic nature of detection that generally applies to small numbers of spores of C. botulinum. In relation to the contamination of food by pathogenic microorganisms, the role of between-batch and within-batch variability has been considered in detail by Commeau et al. (23).

A practical representation is based on log-normal variability for the batch concentration of C. botulinum

$$p\left(s|\mu ,\sigma \right)=\frac{1}{\sqrt{2\pi }}\frac{1}{s\sigma }e^{\frac{{-(\ln\left(s\right)-\text{μ})}^{2}}{{2\text{σ}}^{2}}}$$

where the parameters μ and σ are the means and the standard deviations of ln(*s*). These parameters are related to <*s*> and σ*_s_*, the mean and standard deviation of *s* [i.e., <*s*> = *e*^μ+σ^2^/2^ and σ*_s_/*<*s*> = $\sqrt{e^{\sigma^{2}}-1}$]. The variability of the spore concentration accounts for different loads in distinct batches of materials. The log-normal form of the variability corresponds to many multiplicative effects on contamination, but in realistic situations where the origin of contamination is simply unknown, it is also a convenient method of expressing the variation of a numerical quantity over several orders of magnitude. We choose to parameterize the variability of the spore concentration by the mean value (location), <*s*> (per kilogram), and by the coefficient of variation (spread), σ*_s_/*<*s*>. This parameterization is particularly convenient for considering spore loads in food materials for two reasons: first, when the load is small, the mean value of concentration is more easily interpreted than μ; second, the coefficient of variation is directly related to the standard deviation of the logarithm of the concentration, σ, and is independent of the actual value.

In turn, the parameters of the variability are considered to be uncertain, i.e., each parameter has a single value, but we do not have sufficient information to make a precise estimate. We choose to represent prior belief about the mean spore concentration with a BetaPert uncertainty distribution

$$<s>∼\text{BetaPert}\left({<s>}_{\min},{<s>}_{\mathrm{mod}},{<s>}_{\max}\right)$$

where, for each food category, <*s*>_min_, <*s*>_mod_, and <*s*>_max_ (per kilogram) are the minimum, modal, and maximum values for the mean concentration, respectively. The BetaPert distribution is a unimodal form (a constrained beta distribution) that is traditionally used for representing numeric values within a finite range, but other unimodal distributions would be equally valid. Prior beliefs concerning the coefficient of variation of the spore concentration are represented by a uniform uncertainty distribution

$$\frac{\sigma_{s}}{<s>}\approx \text{Uniform}\left({\left(\frac{\sigma_{s}}{<s>}\right)}_{\min},{\left(\frac{\sigma_{s}}{<s>}\right)}_{\max}\right)$$

where (σ*_s_/*<*s*>)_min_ and (σ*_s_/*<*s*>)_max_ are the limiting values. In the majority of situations, the coefficient of variation for batch concentration is largely unquantified and the uniform distribution represents uninformed beliefs; other featureless distributions are equally valid. A uniform distribution of the coefficient of variation emphasizes the larger values in the corresponding distribution of σ.

We assume that the parameter values for the uncertainty distribution of the mean spore load can be established from the database, and in turn the uncertainty distributions can be considered informative priors in a Bayesian framework. In practice, the database records contain an inseparable mixture of variability and uncertainty. Although there are many ways to evaluate the records, we have used the following consistent scheme for estimating the parameters for the uncertainty distribution of the mean batch concentration. (i) Records were extracted from the database according to material category. (ii) For each record, an MPN measure and an associated standard error for the spore concentration were established. (iii) For each material category, the estimate for <*s*>_mod_ was set as the uniformly weighted average of the MPN values (spores per kilogram). (iv) For each material category, the estimate for <*s*>_max_ was set as the uniformly weighted average of the values for the upper 95% confidence limit for the MPN (spores per kilogram). (v) We fixed <*s*>_min_ as a spore load of zero.

Table 2 indicates parameter values for uncertainty distributions of the mean spore concentration that have been extracted from the collected data set. Each database record includes a number of distinct tests. For example, the 81 shellfish records include 3,874 distinct tests, and the 13 mushroom and fungus records represent 1,410 individual tests.

**TABLE 2:** Prior belief concerning parameter values for uncertainty distribution of mean batch concentrations of nonproteolytic C. botulinum spores^*a*^

Food category	*N*^*b*^	Value^*c*^ (kg^−1^)	
		<*s*>_mod_	<*s*>_max_
Meat	73	7.8	45.9
Fish	574	51.4	268.8
Shellfish	81	145.9	543.3
Cereals	12	8.6	60.7
Plants	91	24.6	171.9
Dairy liquid	9	7.5	54.7
Dairy nonliquid	20	34.0	125.5
Mushroom and fungi	13	228.7	666.8
Herbs and spices	9	27.8	150.0

a The values shown are based on data from the literature.

b *N* is the number of database records.

c <*s*>_mod_ and <*s*>_max_ are the modal and maximum values, respectively, for the mean concentration.

For three material categories, we have compared the beta prior with an alternative construction that uses the average of many log-normal distributions, each constructed from an individual database record (again using derived MPN values). The comparison indicates that the parameterization scheme provides a suitable representation of database information. The informative beta prior consistently gives a smoother distribution over a range similar to that of the composite prior. Although the parameterization scheme truncates the uncertainty distribution of the mean value of the batch concentration at large values, it is important to appreciate that, by including log-normal variability, all nonzero spore concentrations are assigned a finite prior probability.

It is practical to assume prior beliefs about the coefficient of variation for the spore concentration that are independent of the material category. To represent prior belief about variability, we have used single values of (σ*_s_/*<*s*>)_min_ *=* 0.5 and (σ*_s_/*<*s*>)_max_ = 4. The maximum value for the coefficient of variation corresponds to a σ_10_ of ∼0.73, so that it is clear that prior belief for the spore concentration includes the possibility of log-normal variability distributions for which 95% of the probability mass extends over three orders of magnitude [σ_10_ = ln(10)σ is the standard deviation of log_10_(*s*)]. The standard deviation, σ_10_, varies at a very low rate (logarithmically) with the coefficient of variation, so that prior beliefs are relatively insensitive to the choice for (σ*_s_/*<*s*>)_max_. Limpert et al. (24) lists the coefficient of variation for numerous natural populations that occupy this range.

**FIG 1:**
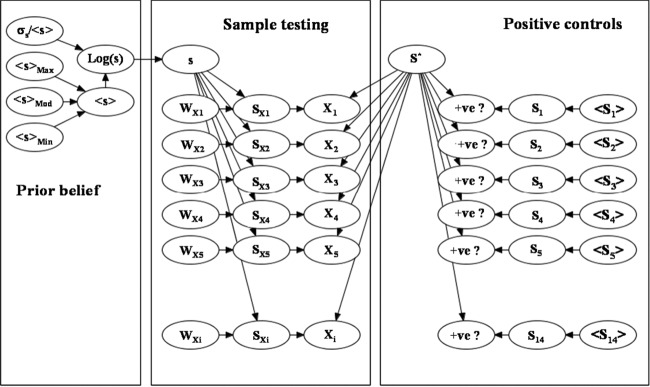
Network representation for a statistical model of spore concentration in food materials. The labeled objects (nodes) represent uncertain quantities, and arrows indicate dependency. In the first panel, <*s*> and σ*_s_* are the means and the standard deviations of the spore concentration, *s* (kg^−1^). In the second panel, *W_Xi_* and *S_Xi_* are the size and the load of the *i*^th^ sample and *X_i_* is the outcome of the *i*^th^ test (positive or negative). In the third panel, S* (number of spores) is the limit of detection, and <*S_i_*> and *S_i_* are the expectation and the actual load in the *i*^th^ control experiment, respectively. The second and third panels extend to include the complete set of samples and controls.

**FIG 2:**
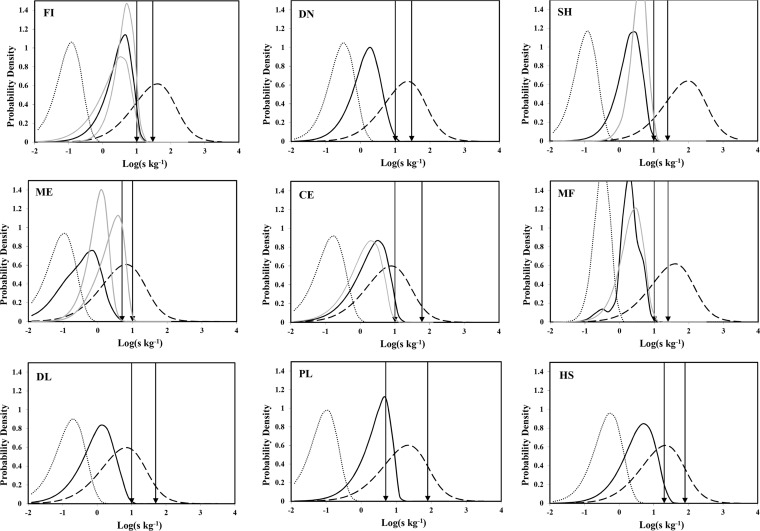
Beliefs concerning the logarithm of the concentration for nonproteolytic C. botulinum spores in nine categories of food materials (fish [FI], meat [ME], dairy liquids [DL], dairy nonliquids [DN], cereals [CE], plant materials [PL], shellfish [SH], mushroom and fungi [MF], and herbs and spices [HS]). Broken lines represent prior beliefs, and full lines represent posterior beliefs. Additional dotted lines indicate the posterior belief given perfect detection of spores, and vertical arrows indicate an approximate 95% confidence interval for the maximum likelihood estimate of the limit of detection. For shellfish, the gray line indicates posterior beliefs following a hypothetical positive result of an additional test. For mushroom and fungi, the gray line indicates posterior belief following a hypothetical case in which two positive test results are absent. In the meat panel, two gray lines indicate spore concentration in 80:20 and 20:80 mixtures of meat and plant material. In the cereal panel, the gray line indicates posterior belief for the spore loads when a conflict, arising from a rare case in the evidence from positive controls, is removed. For fish, two gray lines indicate posterior beliefs for alternative forms of prior information: first when the prior probability for the coefficient of variation of the batch spore load is uniformly distributed in the range of 0.5 to 8, and second when the prior probability for the mean value of the batch spore load is uniformly distributed in the range of 250 to 260 spores kg^−1^.

Parameter uncertainty can be combined with log-normal population variability to give a representation for belief concerning the concentration of nonproteolytic C. botulinum in food materials. A schematic illustration of the structure for the joint probability of the parameters and the variables is illustrated on the left side of Fig. 1. Marginal (prior) beliefs about the logarithm of the concentration of nonproteolytic C. botulinum spores in food materials are illustrated by broken dark lines in Fig. 2. The separation of uncertainty and variability imposed by this quantification scheme is not unique, but it is robust, reproducible, and flexible.

### Tests for spores of nonproteolytic C. botulinum in real food materials.

A total of 483 samples of food materials, obtained from companies involved in chilled food manufacturing in the United Kingdom, were tested for the presence of nonproteolytic C. botulinum spores. The samples were chosen to represent the nine different food categories highlighted during a literature review concerning the natural occurrence of spores in food and, where possible, reflected the significance of each category for United Kingdom chilled food manufacturing. Additionally, the samples were selected to be as diverse as possible, e.g., reflecting different sources, seasons, and species. For example, the 54 samples of fish tested included salmon (18 samples), haddock (15), cod (12), hake (3), coley (3), and monkfish (3). Similarly, the 50 meat samples included chicken (21 samples), beef (16), lamb (5), pork (5), and turkey (3), while the 70 shellfish samples included prawns (48 samples), mussels (10), crab meat (9), and lobster (3). Samples that had been treated in any way that might reduce the spore load were excluded from testing.

An optimized protocol that combines selective enrichment culture with multiplex PCR was used to test the food materials for the presence of nonproteolytic C. botulinum (13). Detection of proteolytic C. botulinum using this protocol is unlikely, as its detection limit is reported as >10^7^ spores kg^−1^ (13). Additionally, type B, E, and F strains each form a unique PCR band, and the type B4 and F6 neurotoxin genes, exclusively present in nonproteolytic C. botulinum types B and F (8, 9), can be identified through sequencing. The PCR test was based on a multiplex PCR method for simultaneous detection of type A, B, E, and F botulinum neurotoxin genes initially developed by Lindström et al. (25) and later modified (13). New primers (forward, 5′-CGGCTTCATTAGAGAACGGATGTCGTGCCAGCTGCATTAA-3′; reverse, 5′-TAACTCCCCTAGCCCCGTATGCCGGATCAAGAGCTACCAAC-3′) were included for the internal amplification control to give an 800-bp product. Each food material sample was 100 to 200 g, except for dried porcini mushrooms (which absorbed water to reach ∼200 g wet weight) and dried herbs and spices (for which the sample size was reduced to 50 g). For samples that appeared positive in the PCR test, the toxin gene PCR product was purified and then sequenced. The gene sequence was confirmed as that of the C. botulinum neurotoxin gene through a BLAST search of all available sequence data.

There is evidence that many herbs and spices possess antimicrobial activity (26, 27). Since there were concerns that herbs and spices could inhibit spore detection, the procedure for preparing these samples, before adding the enrichment medium, was modified. The method used was based on that described by Bianco et al. (28) for determining the prevalence of C. botulinum in fresh and dried chamomile. In this case, 50-g samples of herbs and spices were stirred in 200 ml saline solution for 30 to 60 min to aid the separation of any spores present. The suspensions then were filtered through sterile cotton gauze, and the filtrates were centrifuged (12,000 × *g* for 15 min) to concentrate any spores in the samples. After discarding the supernatants, pellets were resuspended in 10 ml anaerobic saline before adding the enrichment medium.

The detection limit for spores of nonproteolytic C. botulinum was determined for each food material using a protocol essentially as described previously (13). For each food category, 14 sample bottles were prepared from composite foods (some foods used multiple series of 14 bottles; see Table 3). Bottles were inoculated with a mixture of type B (Eklund 2B and Kapchunka B2), type E (Beluga and CB-K-18E), and type F (Eklund 202F and Craig 610) strains of nonproteolytic C. botulinum with target loads of 4 (six replicates), 10 (four replicates), 30 (three replicates), and 100 (one replicate) spores bottle^−1^. The spores were produced using a two-phase cooked meat medium and washed as described previously (29), and nominal inoculum concentrations were determined for each spiking procedure using plate counts (29).

## RESULTS

### The limit of detection for nonproteolytic C. botulinum spores in food materials.

For many bacteria, and particularly for C. botulinum, it is essential to appreciate the significance of negative test results in order to establish quantitative beliefs about spore loads in raw materials. A standard method for estimating the limit of detection in food microbiology is to observe the presence or absence of bacteria or spores, using a prescribed protocol, in a series of test samples with a range of known bacterial loads. We assume that each test is independent and that a well-defined but uncertain load, S* spores, partitions the outcomes (i.e., the test indicates the presence of spores only for loads, *S*, where *S* > S*). The limit of detection, S*, can be estimated (using a maximum likelihood method or a Bayesian approach) from a series of test results (*m*), *i* = 1, which indicate *r_i_*, the number of positives from *N_i_* tests, each with a nominal load, <*S_i_*>. In this approach, the threshold for detection is considered a number of spores rather than as a spore concentration. For a range of volumes, this assumption is consistent with the nature of the experimental protocol used to detect spores. Based on a sample volume, it is possible to transform this detection limit into a concentration value (or a range of values) and then interpret the detection of contamination in natural materials. Details of the maximum likelihood method for the determination of the limit of detection are included in the appendix. Figure 1, right, illustrates schematically how the results of the positive-control tests are combined in a representation of the joint probability to facilitate a Bayesian estimate for the full uncertainty distribution of S* (a Bayesian inference uses a broad uninformative prior distribution for S*).

Results from positive controls in each of nine food categories and maximum likelihood estimates for the detection limit are indicated in Table 3. The results show that, for many food materials, it is possible to detect just a few spores of C. botulinum. The maximum likelihood analyses indicate that estimated detection limits are typically in the range of 1 to 10 spores per test sample, and these values vary across food material categories. The interpretation of the estimated detection limit (which is a discrete load in a particular volume) in terms of limiting concentration (which is a continuous variable) is not always straightforward. However, approximate 95% confidence intervals (CI), based on maximum likelihood analyses, are indicated in Fig. 2 for each food category by two arrows. These ranges indicate the potential influence of new information on beliefs concerning spore loads in food materials.

**TABLE 3:** Results from positive-control tests measuring the presence of spores of nonproteolytic C. botulinum in food materials^*a*^

Food material	Weight (g)	Value for positive control				Detection limit		
		r_1_	r_2_	r_3_	r_4_	S*	s* (kg^−1^)	95% CI
Meat	200	6	4	3	1	1	5	5, 10
Fish	200	4	3	2	1	5	25	20, 30
Fish	100	6	4	3	1	1	10	10, 20
Shellfish	200	4	3	3	1	3	15	10, 25
Cereals	100	5	4	3	0	5	50	30, 60
Cereals	100	6	4	3	1	1	10	10, 20
Cereals	100	6	3	1	1	5	50	40, 60
Plant	200	4	1	0	1	9	45	35, 50
Plant	200	6	4	3	1	1	5	5, 10
Plant	100	2	4	2	1	6	60	40, 80
Dairy liquid	100	6	4	3	1	1	10	10, 20
Dairy liquid	100	5	4	2	1	3	30	20, 50
Dairy nonliquid	100	6	3	3	1	2	20	10, 30
Dairy nonliquid	100	6	3	3	1	2	20	10, 30
Mushroom and fungi	200	3	4	3	1	3	15	10, 25
Herbs and spices	50	5	3	3	1	3	60	20, 80

a The control samples have nominal populations, <*S_i_*> *=* 3.8, 9.6, 30, and 94, with test size (*N_i_*) of 6, 4, 3, and 1, where *i* is 1, 4 and *r_i_* is the number of positive observations. The test size is weight, and the maximum likelihood estimates for the limit of detection are S* spores. The limit of detection is converted to a concentration, s* kg^−1^ (with a 95% confidence interval), based on the sample size.

### Evidence relating to nonproteolytic C. botulinum spore loads in food materials.

Targeted observations provide specific evidence relating to the actual spore loads in food materials, and this evidence can be used to update beliefs about uncertain spore concentrations. Ten egg samples tested negative for nonproteolytic C. botulinum, but only 1/14 corresponding control samples tested positive. A variety of approaches were used to extract DNA from these samples to try to eliminate possible inhibitory components. Since a reasonable limit of detection could not be established, eggs were not considered further. From the remaining 473 tests, 471 samples were negative, indicating spore concentrations below a detection limit that is typically ∼60 spores kg^−1^ (Tables 3 and 4). Two samples of mushrooms (both dried porcini mushrooms) tested positive in the PCR test for the type B neurotoxin gene. Sequencing of the PCR product revealed a 100% match to the type B4 neurotoxin gene of nonproteolytic C. botulinum strains IFR 05/025 and CDC3875 and a 99% match to that of strains Eklund 17B and Eklund 2B (30, 31). Note that strains IFR 05/025 and CDC3875 belonged to a class of European isolates, while other nonproteolytic C. botulinum type B4 strains were from North America (30).

**TABLE 4:** Laboratory tests for the presence of nonproteolytic C. botulinum spores in food materials

Category	Weight (g)	No. of samples		
		Total	Positive	Negative
Meat	200	50	0	50
Fish	200	54	0	54
Shellfish	200	70	0	70
Cereals	100	60	0	60
Plant	200	60	0	60
Dairy liquid	100	46	0	46
Dairy nonliquid	100	37	0	37
Mushroom and fungi^*a*^	200	57	0	57
Mushroom and fungi^*b*^	50	3	2	1
Herbs and spices	50	36	0	36

a Fresh mushrooms.

b Dried porcini mushrooms.

The two samples giving a positive PCR result for C. botulinum toxin genes were examined further. The concentration of spores of nonproteolytic C. botulinum in dried porcini mushrooms was determined using a three-tube MPN method that followed the optimized protocol described earlier, again with appropriate positive controls to estimate the detection limit. The samples were reduced to a fine homogenous grit using a sterile mechanical blender, and 40-g samples were tested at the first level, together with a further five 10-fold dilutions. Three control series were prepared from a porcini mushroom sample that tested negative in the initial tests. They were inoculated at 10^5^, 10^3^, and 10^2^ spores kg^−1^ with the mixed spore suspension used in the positive-control experiments. Both test samples were negative in all MPN tubes, which equated to a spore concentration of less than ∼65 spores kg^−1^ (95% CI, 11 to 548). Therefore, the concentration of spores in these two samples is within the likely range of 60 to 65 spores kg^−1^. Attempts were made to isolate nonproteolytic C. botulinum from the original enrichment cultures by streaking onto plates of Trypticase peptone glucose yeast extract (TPGY) agar containing 10% (vol/vol) egg yolk emulsion and culturing anaerobically at 30°C, but these were unsuccessful.

Assuming that food samples are independent and that in each sample the contamination is representative of spore loads in a particular food category, the test results have a simple statistical interpretation. The number of spores in a test sample is distributed as *S_x_* ∼ Poisson(*sW_X_*), where *s* is the batch concentration of spores and *W_X_* is the sample size. In turn, the probability of a positive result in the *i*^th^ test is expressed as

$$P\left(X_{i}=+\text{ve}|S_{Xi},S^{*}\right)=\theta \left(S_{Xi}-S^{*}\right)$$

where θ is a unit step function. This dependence relationship between the test results and the limit of detection for spores is represented schematically by the central portion of Fig. 1.

### Posterior belief concerning nonproteolytic C. botulinum spore concentrations in food materials.

Extensive evidence from microbiological tests on food samples can be used to update established prior beliefs concerning the spore concentration in food materials. The test results are dominant negative so that, generally, they increase beliefs relating to small batch loads at the expense of beliefs about large loads; however, this change is conditioned by evidence indicating that the limit of detection for spores in food materials is finite. Consistent updated beliefs can be established as Bayesian posterior distributions.

The technical process of Bayesian inference (combining observational evidence with prior beliefs) has become practical for complex information systems because of recent advances in algorithmic and computing power (e.g., see Kjaerulff and Madsen [32]). In complex scenarios it is convenient and instructive to represent the relationships between distinct sources of information as a network structure (where nodes represent variables and arrows depict dependency). The network in Fig. 1 is a structure that highlights the relationship between information about spore loads in food materials and information about microbial tests applied to food samples. In addition, the network structure shows how information about a detection limit (for a test with a prescribed protocol) and the results from positive-control experiments are integrated into the estimation of uncertain spore loads. The results of Bayesian inference for the uncertain batch load, *s*, of spores of nonproteolytic C. botulinum in nine distinct categories of food materials are illustrated by the full lines in Fig. 2. The results were established using a Bayesian belief network implementation of the statistical model (Hugin Expert A/S Aalborg, Denmark) but could be obtained with alternative tools for Bayesian inference, such as Markov chain Monte Carlo simulation. The Bayesian network implementation uses a message-passing algorithm to implement the Bayes theorem consistently within the joint probability of all uncertain variables.

In general, for each food category the posterior belief expresses the impact of dominant-negative tests for nonproteolytic C. botulinum spores. Repeated negative tests make large spore concentrations increasingly unlikely, so that posterior probability is concentrated on loads that are smaller than the limit of detection. Distributions of posterior probability for spore loads are relatively asymmetric (c.f. prior beliefs), reflecting the one-sided influence of the limit of detection. In this respect, posterior beliefs concerning spore loads in materials from the meat category are slightly anomalous, because in this case, all control tests gave a positive result, so there is no evidence to indicate a lower bound for the limit of detection. For clarity, dotted lines in Fig. 2 show, for each food category, posterior beliefs that would correspond to similar test results obtained using a technique with perfect sensitivity (i.e., a limit of detection of S* ∼ 1). For assistance with computations, such as those that might contribute to quantitative risk assessments for C. botulinum hazards, a precise representation of the posterior beliefs in terms of cubic B splines is included in the supplemental material. Some elements of the cumulative probability, representing posterior beliefs for spore concentration (i.e., the probability that the spore concentration exceeds a particular concentration), are indicated in Table 5. For each food category, the probability that the concentration exceeds *s* = 1 spore kg^−1^, given the evidence, is greater than 0.17. In contrast, for all food categories except herbs and spices, the probability that the spore concentration exceeds *s* = 30 spores kg^−1^, given the evidence, is less than 6.1 × 10^−9^. A higher probability for herbs and spices reflects the smaller sample sizes used for microbiological tests (this constraint is imposed by practical considerations). Appreciating this limitation is straightforward, since for test sizes of ∼50 g, negative tests leading to reduced belief only relate to concentrations that exceed ∼20 spores kg^−1^ (this limit would be lower for tests that use larger sample sizes). For herbs and spices, a similar number of negative tests, involving sample sizes of ∼200 g, would give a posterior belief (cumulative probability, *P*) of *P*(*s* > 30 kg^−1^ | {*X_i_ =* -ve, *i* = 1, 36}, *W_X_* *=* 200 g, HS) ≈ 2.4 × 10^−13^, which is in line with the other materials (*X_i_* = -ve indicates a negative result for the *i*th test).

**TABLE 5:** Elements of the cumulative posterior probability for batch spore concentration in food materials^*a*^

Category	*P*(*s* > 1 kg^−1^ | {*X*})	*P*(*s* > 10 kg^−1^ | {*X*})	*P*(*s* > 30 kg^−1^ | {*X*})
Meat	0.17	2.1 × 10^−7^	3.8 × 10^−20^
Fish	0.85	2.1 × 10^−2^	1.9 × 10^−9^
Shellfish	0.79	8.5 × 10^−4^	2.1 × 10^−13^
Cereals	0.72	1.4 × 10^−2^	6.1 × 10^−9^
Plant	0.82	1.1 × 10^−2^	5.3 × 10^−15^
Dairy liquid	0.51	9.2 × 10^−4^	2.6 × 10^−10^
Dairy nonliquid	0.65	1.6 × 10^−3^	9.8 × 10^−10^
Mushroom and fungi	0.87	3.3 × 10^−4^	8.3 × 10^−14^
Herbs and spices	0.84	1.5 × 10^−1^	3.7 × 10^−3^

a [*X*] indicates a complex combination of evidence from systematic surveillance and from positive controls.

Posterior probabilities for the logarithm of the concentration of nonproteolytic C. botulinum spores in food materials represent a consistent combination of prior beliefs with gathered evidence. The top right portion of Fig. 2 (shellfish) illustrates the typical sensitivity of the established posterior belief with respect to evidence. The gray line is a posterior distribution which follows from the addition of evidence from a further hypothetical microbiological test (with test size of 200 g) that gives a positive result. The (hypothetical) positive result reduces beliefs relating to very low loads and increases belief for spore concentrations (*s*) of ∼15 kg^−1^ (which is the concentration that corresponds to a detection limit of S* ∼ 3 spores). Within the belief network representation, probabilities can be combined so that this hypothetical evidence has the probability *P*(*X_71_ =* +ve | {*X_i_* *=* -ve, *i* = 1, 70}, *W_X_* = 200 g, SH) ≈ 5 × 10^−3^.

In Fig. 2, the irregular posterior distribution for spore loads in mushroom and fungi includes additional complexity, because in this case microbiological tests were performed for samples of different sizes. Since for the defined test the limit of detection corresponds to a discrete number of spores, the evidence from samples with sizes of 200 g and 50 g has an impact on different parts of the posterior distribution of spore concentration (i.e., tests that use larger sample sizes can provide evidence that relates to lower spore concentrations). The consistency of Bayesian updating is unaffected by multiple test sizes, and the complex posterior distribution for mushroom and fungi reflects a differential influence of evidence from different sample sizes. It also is important to appreciate that for the statistical model that represents beliefs about spore loads, illustrated in Fig. 1, positive test results can contribute to the development of posterior beliefs about the limit of detection (i.e., positive tests on small test sizes increase beliefs for a small detection limit). In the portion of Fig. 2 that corresponds to mushroom and fungi, a full gray line represents hypothetical posterior beliefs concerning spore loads in mushroom and fungi in the absence of any positive test results. For mushroom and fungi, as expected, beliefs concerning small spore loads are reduced by two positive test results, but at the same time a small number of positive tests causes a small decrease in belief concerning spore loads that are in the tail of the observed limit of detection. This counterintuitive effect corresponds to increased beliefs about lower values for the limit of detection following observations of positive test results for small test sizes (this shift in the limit of detection is not indicated by the arrows in Fig. 2, which are based on a maximum likelihood analysis of the control experiments).

## DISCUSSION

Posterior beliefs concerning concentrations of C. botulinum spores in food materials indicate typical loads that are smaller than many reported in the scientific literature. This shift reflects new evidence from significant numbers of negative results following microbiological tests on food samples combined with detailed evidence concerning the limit of detection. Current beliefs cannot rule out undetected spore loads with concentrations smaller than ∼10 spores kg^−1^, but they provide confidence concerning the small probability for very heavily contaminated food materials.

Posterior beliefs concerning C. botulinum spore concentrations in food materials may contribute to decision-making in relation to food safety. Decision-making under uncertainty is a complex process, and in most cases posterior beliefs about loads are combined with other information in developing safety outcomes. However, the posterior probabilities and their numerical representation, established above, can lead directly to several quantifications with immediate interpretations.

For example, the probability that a particular volume of material, *V*, contains spore numbers, *S*, beyond a particular threshold, *S_0_*, is given by

$$p\left(S>S_{0}|V,\text{food}\;\text{material}\right)=\int_{0}^{\infty }\overset{S\;=\;\infty }{\underset{S\;=\;S_{0}+1}{\sum }}\text{Poisson}\left(S|sV\right)p\left(s\right)ds$$

where *p*(*s*) is the probability density for spore concentration in food material. For dairy nonliquids the probability of exceeding a load of 10 spores in 100 g of material is *p*(*S* > 10| 100 g, DN) ≈ 3 × 10^−10^. This calculation can easily be extended to mixtures of food materials (see the appendix). Based on the numerical representation in the supplemental material, the uncertainty distribution for spore loads in binary mixtures of food materials can be established by simple quadrature (see the appendix). In the portion of Fig. 2 corresponding to meat, two full gray lines represent beliefs concerning spore density for nonproteolytic C. botulinum in simple 80:20 and 20:80 mixtures of meat and plant materials.

For each food material, the development of belief concerning spore concentration involves the assessment of complex multicomponent evidence (i.e., results from many controls and tests). Belief network structures include the facility to measure the internal consistency of complex evidence and make it possible to identify and trace any potential conflicts. A conflict measure is given as

$$\chi \left(\text{ε}\right)=\log_{2}\left(\frac{\prod_{i=1}^{n}p\left({\text{ε}}_{i}\right)}{p\left(\text{ε}\right)}\right)$$

where *p*(ε) is the joint probability of the evidence ε *=* {ε_*i*_, *i* = 1, *n*} that is composed from *n* components ε_*i*_, which have individual probabilities, *p*(ε_*i*_) (32). When pieces of evidence are positively correlated, i.e., not in conflict, we expect the probability of joint evidence to exceed the product of the probabilities for the individual pieces (i.e., one piece of evidence makes another element in the evidence set more likely) so that the logarithm is negative. Alternatively, in conflict situations, we expect χ(ε) to be positive. For seven of the nine food material categories the conflict measure is negative, indicating no significant conflicts in the evidence sets used to develop posterior beliefs about spore loads. The positive test results for mushroom and fungi are not in conflict with the rest of the relevant evidence. However, for cereals and for plant materials the conflict measurements are positive, i.e., 42 and 26, respectively. Within a belief network structure it is possible to examine the origins of conflicts by looking at components of the measure at different nodes of the junction tree (a structure that is complementary to the network itself and facilitates rapid computations concerning elements of joint probability).

For cereals, a single piece of evidence is responsible for the majority of the measured conflict. Conflict is associated with a single anomalous (negative) control result at high nominal spore loads (Table 3). Further, searching within the belief network structure, it is possible to ascertain that the conflict would be explained (and the total conflict measure would become negative) if the actual spore load in the control experiment was less than 7 spores (Fig. 1, right, indicates the position of the actual spore load in control experiments). In this case, the expected load for the control experiment was 100 spores and the actual load is Poisson distributed, so the explanation involves a rare case. Nonetheless, this reasoning is able to explain the conflict. The significance of this rare case is illustrated by a gray line in the cereal portion of Fig. 2, which indicates posterior belief for spore loads in the absence of the spurious control result.

Similarly, for plant materials, conflict is associated with evidence from control experiments, but in this case, conflict is not dominantly associated with a single result (there are many partial conflicts), and it is not possible to develop a hypothesis that explains the conflict as the result of a rare case. For plant materials, conflict suggests that the model describing spore loads does not fully align with the evidence and, more specifically, that this category of food materials is inhomogeneous (i.e., the limit of detection for spores of nonproteolytic C. botulinum has significant variation within this category). In practice, the complex evidence leads to considerable uncertainty in the posterior belief for the limit of detection, which in turn ensures that negative tests are not as effective in reducing beliefs concerning intermediate spore loads.

In this development of belief, some prior distributions are considered to be informative (i.e., they are part of the information supply), but it is important to appreciate that, nonetheless, the observed evidence is dominant in the formation of posterior probability concerning spore loads. In particular, posterior beliefs concerning spore loads in food materials are relatively insensitive to the range used for the uncertainty distribution of the coefficient of variation of batch spore concentration. In Fig. 2, the portion corresponding to fish includes a gray line that represents posterior beliefs based on an alternative prior distribution for the coefficient of variation of batch spore concentration, with σ*_s_/*<*s*> ∼ uniform[0.5,8]. Alternative forms for prior belief concerning the variability of batch spore concentrations, i.e., a uniform uncertainty for σ, may change the shape of the posterior distribution for small loads but do not disturb the sharp decline of probability density in the vicinity of the limit of detection (i.e., there is a strong effect from the observed evidence). It is important to appreciate that, at fixed <*s*>, the mean value of the logarithm of the concentration decreases as the standard deviation, σ, increases. Posterior beliefs concerning spore loads in food materials are more sensitive to prior beliefs concerning the mean value of the batch concentration of spores, but again this effect is significant only at moderate and low concentrations. Within a belief network structure it is possible to calculate exact derivatives that reflect the variation of an output probability with respect to any input probability, called a sensitivity value (33). As an example, for fish, the output probability, *p*(*s* > 30 kg^−1^ | FI, {*X*}), depends on the input probability, *p*(100 kg^−1^ ≤ <*s*> < 110 kg^−1^), an element of the prior uncertainty distribution, but the corresponding sensitivity value is very small, ∼1.0 × 10^−9^ ({*X*} represents the full evidence used to develop a posterior belief about spore concentration in fish). In contrast, posterior (output) probabilities for lower values of spore load, *p*(*s* > 10 kg^−1^ | FI, {*X*}) and *p*(*s* > 1 kg^−1^ | FI, {*X*}), have stronger sensitivity, with sensitivity values of ∼5.9 × 10^−3^ and ∼4.1 × 10^−1^, with respect to the input probability (note that the input probability corresponds to a spore concentration larger than the modal value of <*s*> for fish [Table 2]). In Fig. 2, the portion corresponding to fish includes a gray line that represents posterior beliefs based on a prior distribution for the mean batch concentration of spores, with <*s*> ∼ uniform[250,260] spores per kilogram positioned around the 95th percentile of the informative prior. This distribution supports the conclusions based on the measured changes in sensitivity values obtained across the range of spore concentration values. A consistent sensitivity assessment is obtained by similar tests across all nine material categories.

Most active surveillance operations, such as observations of spore loads in samples of food materials, can be extended in many different ways, but invariably this incurs additional costs. For a structured belief system it is practical to evaluate the potential impact of additional findings using a value-of-information approach; hence, it may be possible to direct the use of new resources. For the posterior distribution of spore load, established for each food material, it is possible to evaluate an “information entropy” as the expectation of −log(*p*(log(*s*)). The entropy represents the disorder associated with the distribution of log(*s*) and quantifies the information deficit that exists for current beliefs (information entropy is widely used to quantify uncertainty in probabilistic scenarios). In turn, a decrease of entropy following new findings quantifies the value that can be attributed to new information and directs choices for prospective investigations (even though the results of new investigations remain uncertain). The change in the entropy of log(*s*) following a new (single) observation for another variable is called mutual information or the value of information; this quantity can be computed easily within a belief network structure (32). For our nine food materials the entropy of the posterior distribution of log(*s*) was in the range of 1.8 to 2.4, and the entropy reduction (value of information), corresponding to additional tests on food samples or additional control experiments, is indicated in Table 6. It is clear that for four of the food materials (meat, fish, shellfish, and mushroom and fungi), additional control experiments involving expected loads of <*s*> ≈ 4 are the most valuable source of new information. For the other materials, testing additional food samples (with a sample size of 200 g) has the most value (note that for all food materials additional control tests with large expected numbers of spores have very little value, i.e., there is a strong belief that the result is already known). Unsurprisingly, additional tests for spores in 200-g samples of herbs and spices represent the most valuable source of new information relating to C. botulinum loads in foods (however, these tests will require additional method development). In contrast, for mushroom and fungi, further controls using small expected numbers of spores represent the most valuable way to extend the quantification.

**TABLE 6:**
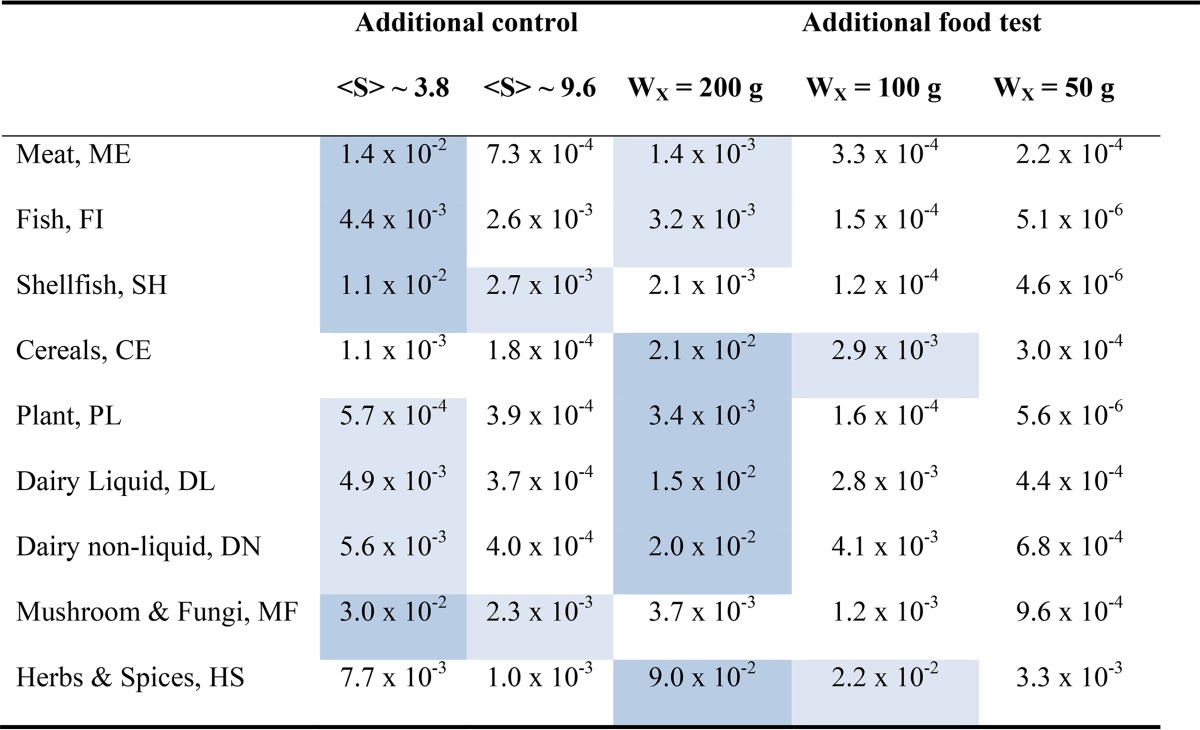
Value of information calculated for new findings in relation to the posterior probability for the logarithm of the spore load in nine food materials^*a*^

a New findings from tests of independent food samples or from control experiments. Values represent mutual information, and for each material the most valuable (dark shading) and second most valuable (pale shading) findings are highlighted.

An appreciation of this model for nonproteolytic C. botulinum spore loads in food materials should include, in addition to the evaluation of numerical sensitivities, a clear picture of underlying assumptions that relate to the nature of the information supply. In particular, the development of belief concerning spore loads is based on a strong assumption that the sources of evidence (i.e., samples of food materials) are independent and that these sources are representative of the model variables (i.e., food samples are representative of the associated [homogeneous] food category). Crucially, this development assumes that the concentrations of spores in food materials behave in a regular fashion and can be represented by smooth, unimodal probability distributions. It is important to appreciate that the nine food categories used in this development are not exhaustive, so this construction cannot be used to represent belief concerning common food materials such as honey or egg.

The posterior probability distributions for nonproteolytic C. botulinum spore concentrations in foods provide a robust and flexible representation of accumulated information that is relevant for consideration of botulism hazards. The Bayesian scheme used to develop beliefs attempts to minimize the influence of models and to highlight the impact of gathered evidence. Numerical representations facilitate the use of extensive information about spore loads in quantitative risk assessments and in decision support processes, and analysis of sensitivities supports ongoing developments or translation toward other objectives.

## APPENDIX

### Maximum likelihood estimation of the limit of detection for spores of C. botulinum in food materials.

The limit of detection, S*, can be estimated from a series of test results, *i* = 1, *m*, which indicate *r_i_*-positive results from *N_i_* tests, each with a nominal load of <*S_i_*>.

We assume that the actual loads, *S*, in the test materials are Poisson distributed with the expectation <*S_i_*>. In a test with nominal load <*S_i_*>, the probability that the actual load exceeds the detection limit is

$$p\left(S>S^{*}|<S_{i}>,S^{*}\right)=q_{i}=1-\overset{S^{*}}{\underset{S=0}{\sum }}\frac{{<S_{i}>}^{S}}{S!}e^{-<S_{i}>}$$

In turn, the likelihood of the test is given as

$$P\left(\left\{r_{i}\right\}|\left\{N_{i}\right\},<S_{i}>,S^{*}\right)=\overset{m}{\underset{i=1}{\prod }}\text{Binomial}\left(N_{i},r_{i},q_{i}\right)$$

where each test at level *i* is considered a Bernoulli trial. The logarithm of the likelihood has *m* contributions and can be maximized with respect to the threshold value S*.

$$\ln\left[P\left(\left\{r_{i}\right\}|\left\{N_{i}\right\},<S_{i}>,S^{*}\right)\right]=\text{Constant}+\overset{m}{\underset{i=1}{\sum }}\left[r_{i}\ln\left(q_{i}\right)+\left(N_{i}-r_{i}\right)\ln\left(1-q_{i}\right)\right]$$

The constant, which depends only on the data and not the threshold, is irrelevant for maximum likelihood determination. The analysis implies a single test volume, *v*, and a corresponding threshold load, S*, but could be extended to heterogeneous tests.

A confidence interval can be established from the likelihood ratio (difference in log likelihood around the maximum value). The ratio

$$\gamma =-2\left[\ln\left(P\left(\left\{r_{i}\right\}|\left\{N_{i}\right\},<S_{i}>,S\right)\right)-\ln\left(\left(P\left\{r_{i}\right\}|\left\{N_{i}\right\},<S_{i}>,S^{*}\right)\right)\right]$$

is assumed to be distributed as χ^*2*^ with 1 df, so that γ > χ^2^_(α, 1)_ defines a 100 × (1 − α) percent confidence interval.

### Spore populations in mixtures of food materials.

For two independent homogeneous materials with batch spore concentrations *s*_1_ and *s*_2_ (kg^−1^) and volumes *V*_1_ and *V*_2_ (kg), the conditional probability for the logarithm of the density of a mixture is

$$p\left(\text{log}\left(s\right)|\text{log}\left(s_{1}\right),\text{log}\left(s_{2}\right),V_{1},V_{2}\right)=\delta \left(\text{log}\left(s\right)-\text{log}\left(\frac{V_{1}s_{1}+V_{2}s_{2}}{V_{1}+V_{2}}\right)\right)$$

where δ is a Dirac delta function and *s* > α*s*_1_, where α = *V*_1_/(*V*_1_ + *V*_2_) is a partial volume. Marginalization leads to

$$p\left(\text{log}\left(s\right)\right)=\int_{-\infty }^{\infty }\frac{s}{s-\alpha s_{1}}p\left(\text{log}\left(s_{1}\right)\right)p\left[\text{log}\left(s_{2}\right)=\text{log}\left(\frac{s-\alpha s_{1}}{1-\alpha }\right)\right]d\text{log}\left(s_{1}\right)$$

Similarly, the total number of spores, *S*, in a mixture is the sum of the contributory numbers *S*_1_ and *S*_2_ in volumes *V*_1_ and *V*_2_. Individually, *S*_1_ and *S*_2_ are Poisson distributed with parameters *s*_1_*V*_1_ and *s*_2_*V*_2_, so the cumulative probability of the total number can be computed as a sum

$$P\left(S_{1}+S_{2}>S|s_{1},s_{2},V_{1},V_{2}\right)=P\left(S_{1}>S|s_{1}V_{1}\right)+\overset{S}{\underset{i=0}{\sum }}P\left(S_{1}=i|s_{1}V_{1}\right)P\left(S_{2}>S-i|s_{2}V_{2}\right)$$

where, on the right, all of the probabilities are Poisson. To include uncertainty from batch concentrations, this expression has to be integrated over both *s*_1_ and *s*_2_, with appropriate density, but the form of this expression ensures that double integrals are not required.

## Supplementary Material

Supplemental material
