# Supplementary material for: Quantification of Nonproteolytic Clostridium botulinum Spore Loads in Food Materials
Source: Appl Environ Microbiol. 2016 Mar 7;82(6):1675–85. doi: 10.1128/AEM.03630-15 (PMC4784027; doi:10.1128/AEM.03630-15)
Supplement: Supplemental material [file supp_82_6_1675__index.html]

Supplemental material 

# Quantification of non-proteolytic *Clostridium botulinum* spore loads in food materials

## Supplemental material

- Supplemental file 1 -

  Supplemental text and equations and ﻿﻿﻿﻿﻿coefficients for cubic B-spline interpolation of the logarithm of the posterior probability density for the spore concentration in food materials (supplemental table).

  PDF, 544K
- Supplemental file 2 -

  Reference information.

  XLSX, 20K
- Supplemental file 3 -

  Variable descriptions.

  XLSX, 11K
- Supplemental file 4 -

  Distribution data.

  XLSX, 190K
