# Supplementary material for: Quantification of Nonproteolytic Clostridium botulinum Spore Loads in Food Materials
Source: Appl Environ Microbiol. 2016 Mar 7;82(6):1675–85. doi: 10.1128/AEM.03630-15 (PMC4784027; doi:10.1128/AEM.03630-15)
Supplement: Supplemental material [file AEM.03630-15_zam999116982so1.pdf]

Quantification of non-proteolytic *Clostridium botulinum* spore loads in food materials

Gary. C. Barker, Pradeep K. Malakar, June Plowman and Michael W. Peck*

Representation of posterior probability distributions using cubic B-splines

The posterior probability distributions for the logarithm of spore concentration in food materials have complex forms that can, most economically, be represented by cubic B-splines (e.g. Press WH., Teukolsky SA, Vettering WT, Flannery BP. 1986. Numerical recipes in Fortran. Cambridge University Press, Cambridge.). These forms provide the necessary facility for secondary computation such as marginalization or convolution etc.

The logarithm of the posterior probability is chosen as the interpolation variable and splines are based on 21 evenly spaced points beginning with $x_0 = -1.9$ and with an increment $\Delta x = 0.2$ (where $x = Log(s)$ and s kg^{-1} is the spore concentration). For each food category the spline is given as

$$S_3(x) = \sum_{i=-1}^{21} a_i \, B_i(x)$$

with $B_i(x) = B_0(x - i\Delta x)$ and

$$B_0(x) = \begin{cases} 0 & x \le x_0 - 2\Delta x \\ (2\Delta x + x - x_0)^3/6 & x_0 - 2\Delta x \le x \le x_0 - \Delta x \\ 2\Delta x^3/3 - (x - x_0)^2(2\Delta x + x - x_0)/2 & x_0 - \Delta x \le x \le x_0 \end{cases}$$

$$\begin{cases} 2\Delta x^3/3 - (x - x_0)^2(2\Delta x - x + x_0)/2 & x_0 \le x \le x_0 + \Delta x \\ (2\Delta x - x + x_0)^3/6 & x_0 + \Delta x \le x \le x_0 + 2\Delta x \\ 0 & x_0 + 2\Delta x \le x \end{cases}$$

The coefficients a_i *with* $i = 0, 20$ are given, for each food category in the table below and $a_{-1} = 2a_0 - a_1$, $a_{21} = 2a_{20} - a_{19}$.

Table: Coefficients for cubic B-Spline interpolation of the logarithm of the posterior probability density for the spore concentration in food materials – Meat (ME), Fish (FI), Dairy liquid (DL), Dairy non-liquid (DN), Cereals (CE), Plants (PL), Shellfish (SH), Mushroom & Fungi (MF), Herbs & Spices (HS).

**B-Spline coefficient**	**Food Material**								
	**ME**	**FI**	**DL**	**DN**	**CE**	**PL**	**SH**	**MF**	**HS**
$\mathbf{a_{0}}$	-149.6	-369.8	-228.5	-321.1	-270.9	-324.8	-407.5	-610.7	-346.3
$\mathbf{a_{1}}$	-122.4	-333.8	-200.2	-279.9	-241.0	-293.0	-360.8	-511.4	-311.7
$\mathbf{a_{2}}$	-96.5	-295.8	-171.7	-239.4	-212.6	-260.0	-315.1	-416.3	-276.4
$\mathbf{a_{3}}$	-73.6	-258.1	-143.7	-200.4	-183.0	-227.6	-269.8	-326.3	-241.0
$\mathbf{a_{4}}$	-54.9	-220.9	-117.1	-163.1	-155.0	-195.3	-225.5	-244.7	-206.8
$\mathbf{a_{5}}$	-41.6	-185.4	-92.1	-128.6	-128.2	-164.0	-182.9	-174.9	-174.1
$\mathbf{a_{6}}$	-33.1	-151.3	-69.4	-96.8	-103.2	-134.6	-142.1	-125.3	-143.2
$\mathbf{a_{7}}$	-25.5	-119.0	-49.0	-68.1	-80.2	-106.3	-103.7	-97.8	-114.4
$\mathbf{a_{8}}$	-16.0	-88.8	-31.1	-42.6	-59.3	-80.4	-68.2	-132.9	-88.2
$\mathbf{a_{-9}}$	-12.9	-60.9	-16.7	-20.4	-40.9	-56.4	-36.5	-82.8	-64.2
$\mathbf{a_{10}}$	-28.9	-35.5	-8.1	-3.5	-25.2	-34.9	-9.9	21.9	-43.3
$\mathbf{a_{11}}$	-78.5	-13.2	-9.8	3.0	-12.9	-15.8	9.7	38.2	-25.6
$\mathbf{a_{12}}$	-123.0	4.3	-29.6	-10.0	-5.5	0.3	11.2	-18.4	-12.8
$\mathbf{a_{13}}$	-239.9	11.0	-57.9	-46.0	-10.2	9.7	-10.7	-5.4	-7.4
$\mathbf{a_{14}}$	-406.2	-10.2	-129.7	-92.1	-31.6	-4.7	-87.7	-115.8	-11.4
$\mathbf{a_{15}}$	-709.2	-96.5	-271.3	-248.8	-119.8	-118.8	-265.4	-316.9	-28.4
$\mathbf{a_{16}}$	-1244.2	-340.1	-537.8	-479.1	-320.6	-467.8	-629.9	-704.3	-79.8
$\mathbf{a_{17}}$	-2173.9	-865.7	-1010.0	-941.7	-798.1	-1534.2	-1374.9	-1453.0	-175.9
$\mathbf{a_{18}}$	-3953.8	-2060.8	-1948.0	-1843.6	-1809.6	-3435.6	-2702.3	-2624.0	-381.6
$\mathbf{a_{19}}$	-6833.3	-4141.6	-3487.2	-3327.7	-3639.9	-8917.9	-5601.7	-5554.0	-724.8
$\mathbf{a_{20}}$	-13157.4	-9116.2	-7079.8	-6693.5	-8155.0	-13250.0	-7875.0	-7875.0	-1560.8
